# Supplementary material for: A data-driven interpretation of the stability of organic molecular crystals
Source: Chem Sci. 2023 Jan 16;14(5):1272–85. doi: 10.1039/d2sc06198h (PMC9891366; doi:10.1039/d2sc06198h)
Supplement: SC-014-D2SC06198H-s001 [file SC-014-D2SC06198H-s001.pdf]

Electronic Supplementary Material (ESI) for Chemical Science.
This journal is © The Royal Society of Chemistry 2022

Supplementary Information: A data-driven interpretation of the stability of organic molecular crystals

Rose K. Cersonsky,*[a] Maria Pakhnova,[a] Edgar A. Engel,[b] and Michele Ceriotti[a]

A Constructing the Datasets

A.1 Dataset Curation

We start with a dataset containing approximately 10'600 ground-state, geometry-optimized configurations originally used for training (10'000) and testing (604) a model for predicting NMR chemical shieldings for organic molecular crystals[1]. These molecular crystals contain 14 of the most common chemical species, and their energies have already been computed using the protocol described in Sec. A.2. For our study, we selected all H, C, N, O, and S-containing molecular crystals composed of non-polymeric, neutrally-charged molecules and less than 200 atoms per unit cell. This resulted in 2'707 and 551 such crystals for training and testing, corresponding to 3'242 and 628 molecules, respectively. A similar screening protocol was applied to the ethenzamide co-crystals summarized in Appendix A.4. All data and workflows were managed by the *signac* and *signac-flow* packages[2,3].

Reducing to H, C, N, O, S First, we eliminated all structures containing chemical species other than H, C, N, O, and S. We have limited our dataset to these species to maximize the diversity of our dataset while minimizing the size of our SOAP representation (the length scales with $\mathcal{O}(n_{species}^2)$). This resulted in the largest reduction of the overall dataset, from roughly 10'000 to 3'800 crystals.

Separating the Crystals We then separated each crystal into its molecular constituents. First, we computed radial distribution functions, combining the data from all structures in a single histogram for each pair of chemical species. We determined the cutoff distance for covalent bonds as the first near-zero minimum after the first neighbor peak. We computed a supercell consisting of 3^3-7^3 repeat unit cells for each crystal to screen for polymers.

Determining the Set of Unique Molecules Finally, we determined the irreducible set of constituent molecules by identifying identical/redundant molecules based on the similarity of their SOAP features. In practice, a similarity kernel $K_{mm'} = \mathbf{x}_m \cdot \mathbf{x}_{m'}$ was constructed by computing the similarity of each pair of molecules m and m' and iteratively removing molecules $m' > m$, for which $K_{mm'} > \epsilon$. For the later step of identifying the molecular motifs, we were careful to index the location of each atom in the original crystal within the constituent molecules. .

Eliminating Charged Molecules We computed the molecular charges from density-functional tight-binding (DFTB) calculations, using the DFTB+ package[4] and the Third-Order Parameterization for Organic and Biological Systems (3OB)[5,6] to perform a Γ-point calculation for each crystal structure. Structures containing molecules carrying an absolute charge greater than 0.5e were eliminated. We also eliminated those crystals with common zwitterionic moieties such as NH_3^+ or COO^-. These steps ensure that *for the given dataset* the lattice energies are defined unequivocally.

A.2 Relaxing Molecular Geometries

Quantum-Espresso Parameterization To ensure that the resultant geometries and energies are consistent and comparable to those obtained for the crystal structures in Cordova *et al.*[1], the geometry optimizations for the molecules were performed using the same computational parameters using Quantum Espresso[7]. These parameters were as follows: the PBE exchange-correlation functional[8], the D2 dispersion correction[9], ultrasoft pseudopotentials with GIPAW reconstruction[10,11], and an equivalent plane-wave energy cutoff of 60 Ryd. We converged the energies within 1E-4 Ryd and forces below 1E-3 Ryd/Bohr, respectively. Furthermore, we compute the binding energy based on the lowest energy conformer represented in the dataset, ensuring comparability between crystals and co-crystals of similar stoichiometries without needing to obtain the global minimum conformation of each molecule.

Simulation Boxes for Molecules *in vacuo* To determine the simulation boxes appropriate to describe the molecules *in vacuo*, we performed Γ-point calculations. We converged the results with respect to the size of the simulation cell and, *e.g.*, the

[a] Laboratory of Computational Science and Modeling (COSMO), École Polytechnique Fédérale de Lausanne, Lausanne, Switzerland
[b] TCM Group, Trinity College, Cambridge University, Cambridge, UK
* Present address: Rose.Cersonsky@wisc.edu

separation between the periodic images of the molecules. Based on calculations using a variety of simulation cell sizes for 20 different molecules, we determined a minimum vacuum padding of 2-4 times the largest dimension of the molecule proved sufficient to converge the resultant molecular energies. We confirmed these results against those obtained using Martyna-Tuckerman electrostatic decoupling[12].

A.3 Properties of the Resulting Dataset

Properties of the resulting dataset are given in Fig. S1, with an inset denoting the correlation of a given property with the error in regressing the lattice energy (ϵ_δ) given the best-performing model reported in the text. Red curves and lines denote the testing set, and black curves and lines are the training set.

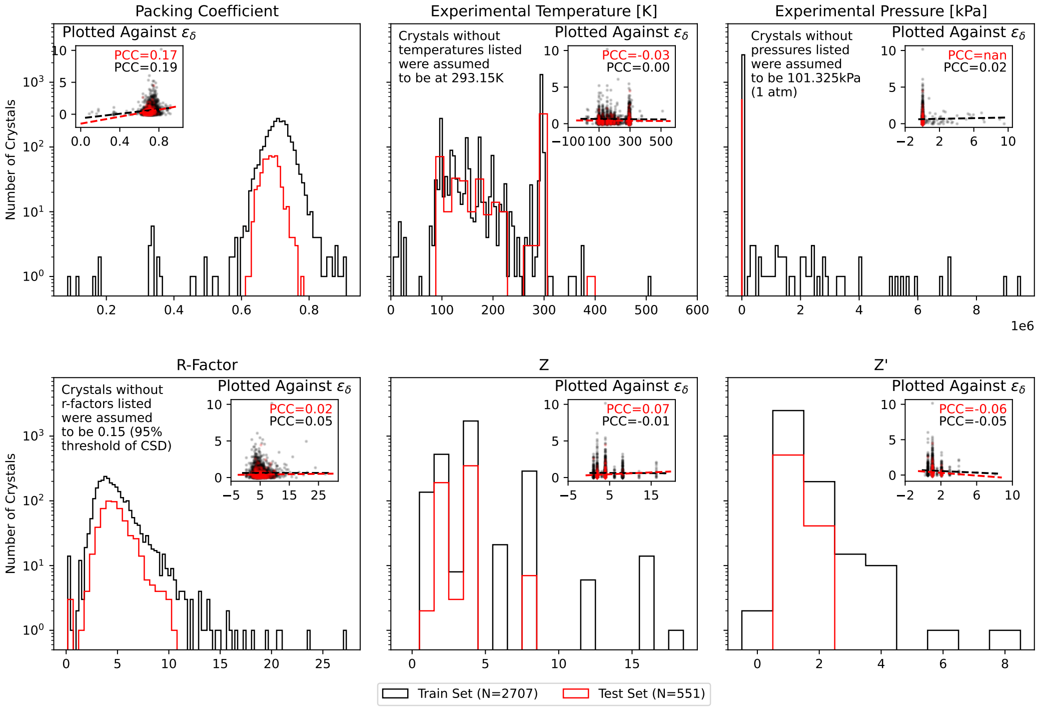

Fig. S1 Analysis of the structures contained in this dataset. In each panel, we show the histogram of a structural parameter across the training set (black) and testing set (red). For parameters not reported in the original data entry, we have assumed ambient conditions, as noted in each panel. In the insets, we show the relationship between these parameters and the error in regressing the binding energy using the remnant model described in the text, ϵ_δ to show that there is the relationship between these parameters and the regression performance.

Of the 3'258 total crystals, 23 (0.7%) are shown to be unstable (*e.g.*, $\Delta_c > 0$) for the reference DFT method at ambient conditions. The majority of these are structures were experimentally determined at high pressure (CSD Refs. VOFVAN23[13], JAYDUI06[14], PHENAN14[15]), low temperature (HAMFEJ[16], ZZZEEU05[17], EHAJUS[18], NTSALA[19], ZAQFAB[20], IDUWEJ[21], UWUFAV[22], UVOMIC[23], LAGMUC[24], GAFYES[25], PUYVAG[26], NUZGOG[27], NSBTOA[28]), or have high r-factors (QNACRD03[29]). From visual analysis, the remaining six crystals (LINJUN[30], XOSBIS[31], TETYOH[32], DBANQU[33], NAPDCX[34], and DUPLUV[35]) appear to have kinetically trapped molecular components, with a large difference between the crystallized molecular geometry and the dilute gas molecular geometry. This can be due to many reasons, but we reason that this is due to small strains induced by the reported lattice parameters, as computing the variable cell relaxations results in a marginally different crystal that has a negative lattice energy.

Table S1 Summary of Crystals with $\delta_c > 0$

	n_c	δ_c [kJ/mol]	Packing Coeff.	R-Factor [%]	Exp. Temp. [K]	Exp. Press. [GPa]	Z	Z'
VOFVAN23 [13]	52	0.0430	0.7760	7.45	295.	1.	2	1
HAMFEJ [16]	68	0.2930	0.7440	3.87	100.	–	4	1
ZZZEEU05 [17]	68	0.3180	0.7580	7.89	20.	–	4	1
LINJUN [30]	68	0.3990	0.7380	4.10	–	–	4	1
EHAJUS [18]	30	0.4420	0.7450	3.18	150.	–	2	0
XOSBIS [31]	60	0.4580	0.7190	2.90	296.	–	4	1
NTSALA [19]	68	1.1170	0.7520	4.90	108.15	–	4	1
ZAQFAB [20]	72	2.0340	0.7090	7.15	123.	–	4	1
TETYOH [32]	80	2.1780	0.7020	4.84	293.	–	4	2
IDUWEJ [21]	30	3.4640	0.72	4.40	123.20	–	2	0
JAYDUI06 [14]	44	3.4890	0.8570	7.64	295.	5.93	4	1
UWUFAV [22]	104	3.7560	0.7650	11.39	100.	–	4	1
DBANQU [33]	144	3.8130	0.75	9.65	–	–	4	1
NAPDCX [34]	48	3.8580	0.7580	8.20	–	–	2	1
UVOMIC [23]	60	4.0790	0.7770	5.43	125.	–	1	0
LAGMUC [24]	16	4.2590	0.7330	2.02	100.	–	2	0
DUPLUV [35]	132	4.8510	0.7410	4.20	–	–	4	1
GAFYES [25]	112	5.67	0.7440	5.88	150.	–	4	1
PUYVAG [26]	14	5.9990	0.7290	4.90	173.	–	1	0
PHENAN14 [15]	96	6.0820	0.7540	11.30	293.	0.70	4	1
NUZGOG [27]	16	8.4160	0.7430	4.26	100.	–	1	0
QNACRD03 [29]	72	9.2720	0.7550	20.60	–	–	2	1
NSBTOA [28]	80	20.9550	0.8150	9.	153.15	–	4	1

A.4 Ethenzamide Co-Crystals

For our case study later in the text, we have curated a set of ethenzamide co-crystals from the Cambridge Structure Database, as summarized in Table S2. We have screened and computed energies using the exact protocols as in A.1 - A.2.

Table S2 Summary of Ethenzamide Co-crystals

	Co-former	n_c	E_c [Ryd]	δ_c [kJ/mol]	Packing Coeff.	R-Factor [%]	Exp. Temp. [K]	Z	Z'
VAKTOS [36]	Ethylmalonic Acid	80	-920.76	-6.74	0.69	4.58	110.0	2	1
ORIKOR [37]	2-NBA	80	-1015.04	-5.98	0.69	6.19	296.0	2	1
VAKTOS01 [36]	Ethylmalonic Acid	160	-1841.51	-6.71	0.68	9.62	110.0	4	1
JIFHAK [38]	2,4 DHBA	160	-1922.96	-6.13	0.65	5.47	296.0	4	1
REHSUU [39]	4-hydroxybenzoic Acid	156	-1756.28	-6.32	0.72	5.98	110.0	4	1
QULLUF [40]	Gentisic Acid (2,5 DHBA)	160	-1922.94	-6.41	0.7	7.63	110.0	4	2
REHTAB [39]	Fumaric Acid	58	-671.35	-6.78	0.69	7.63	110.0	2	1
ORILAE [37]	2,4-DNBA	168	-2470.77	-7.43	0.69	4.03	296.0	4	1
WUZJUX [41]	3,5 DNBA	84	-1235.34	-5.97	0.63	8.04	110.0	2	1
VUHFIO01 [42]	Saccharin	160	-2129.21	-6.18	0.69	5.95	110.0	4	1
VUHFIO [42]	Saccharin	80	-1064.59	-5.91	0.71	3.81	110.0	2	1
WUZJEH [41]	3,5 DNBA, Dioxane	98	-1373.7	-5.97	0.72	7.4	160.0	2	1
WUZJOR [41]	3,5 DNBA	84	-1235.33	-5.87	0.61	6.26	110.0	2	1
QULLUF02 [40]	Gentisic Acid (2,5 DHBA)	160	-1922.9	-6.06	0.72	6.19	110.0	4	1
ODIDEN01 [43]	3,5 DHBA and H2O	160	-2152.0	-8.86	0.7	6.62	296.0	2	1
WUZHOP [41]	3,5 DNBA	168	-2470.72	-6.32	0.72	5.44	110.0	4	1
ORIKIL [37]	Gallic acid	128	-1499.15	-6.49	0.65	3.82	296.0	2	1
TIWPIB [44]	Glutaric Acid	160	-1841.74	-8.03	0.67	4.88	293.0	4	1
WUZKAE [41]	3,5 DNBA	84	-1235.34	-5.97	0.64	6.34	110.0	2	1
REHSAA [39]	Salicylic Acid	156	-1756.29	-5.74	0.71	7.31	100.0	4	1
WUZJIL [41]	3,5 DNBA, Dioxane	98	-1373.7	-5.98	0.72	5.69	110.0	2	1
ODICUC [45]	2,4 DHBA	160	-1922.93	-5.95	0.69	4.49	296.0	4	1
QULLUF01 [40]	Gentisic Acid (2,5 DHBA)	160	-1922.94	-6.43	0.7	12.33	110.0	4	1
WUZKEI [41]	3,5 DNBA	84	-1235.33	-5.9	0.63	5.47	110.0	2	1
ORILEI [37]	3-toluic acid	164	-1644.76	-5.53	0.67	3.91	296.0	4	1
REHSII [39]	Vanillic Acid	172	-1977.88	-6.04	0.7	7.37	193.0	4	1
ORIKUX [37]	3-NBA	160	-2030.17	-6.03	0.69	5.42	296.0	4	1
FENQEX [43]	Gentisic Acid (2,5 DHBA)	160	-1922.94	-6.41	0.7	4.9	296.0	4	2
WUZHOP01 [41]	3,5 DNBA	84	-1235.34	-5.92	0.72	7.62	110.0	2	1

B Methods

B.1 SOAP Hyperparameters

SOAP descriptors were generated using the Librascal library[46] (commit `6f7a4002`) using the following hyperparameters:

- `max_radial`: 8
- `max_angular`: 4
- `interaction_cutoff`: 7.0
- `gaussian_sigma_constant`: 0.3
- `cutoff_smooth_width`: 0.5
- `soap_type`: `"PowerSpectrum"`
- `gaussian_sigma_type`: `"Constant"`
- `radial_basis`: `"GTO"`
- `cutoff_function_type`: `"RadialScaling"`
- `cutoff_function_parameters`: `{rate: 1.5, exp: 3.0, scale: 2.0}`

As tuning these hypers could favor one representation over another, we chose cutoff and scaling parameters to be consistent with the chemical geometry, noting that changing these parameters within this range has minimal effect on the errors. We determined the number of radial and angular channels (corresponding to the “resolution” of the descriptors) by balancing the number of features in each feature vector with the in-sample error of δ_c. The resulting SOAP vectors included 3-body correlations for each atomic neighborhood up to 7Å, weighting neighbor contributions with a radial scaling procedure introduced in Willatt *et al.*[47].

B.2 Filtering the Environmental Contributions

As noted in the main text, we applied a filtering scheme to the estimated contributions of each atomic environment. This technique reduces the number of extreme contributions attributed to any given environment, as shown in the changes in distribution in Fig. S2.

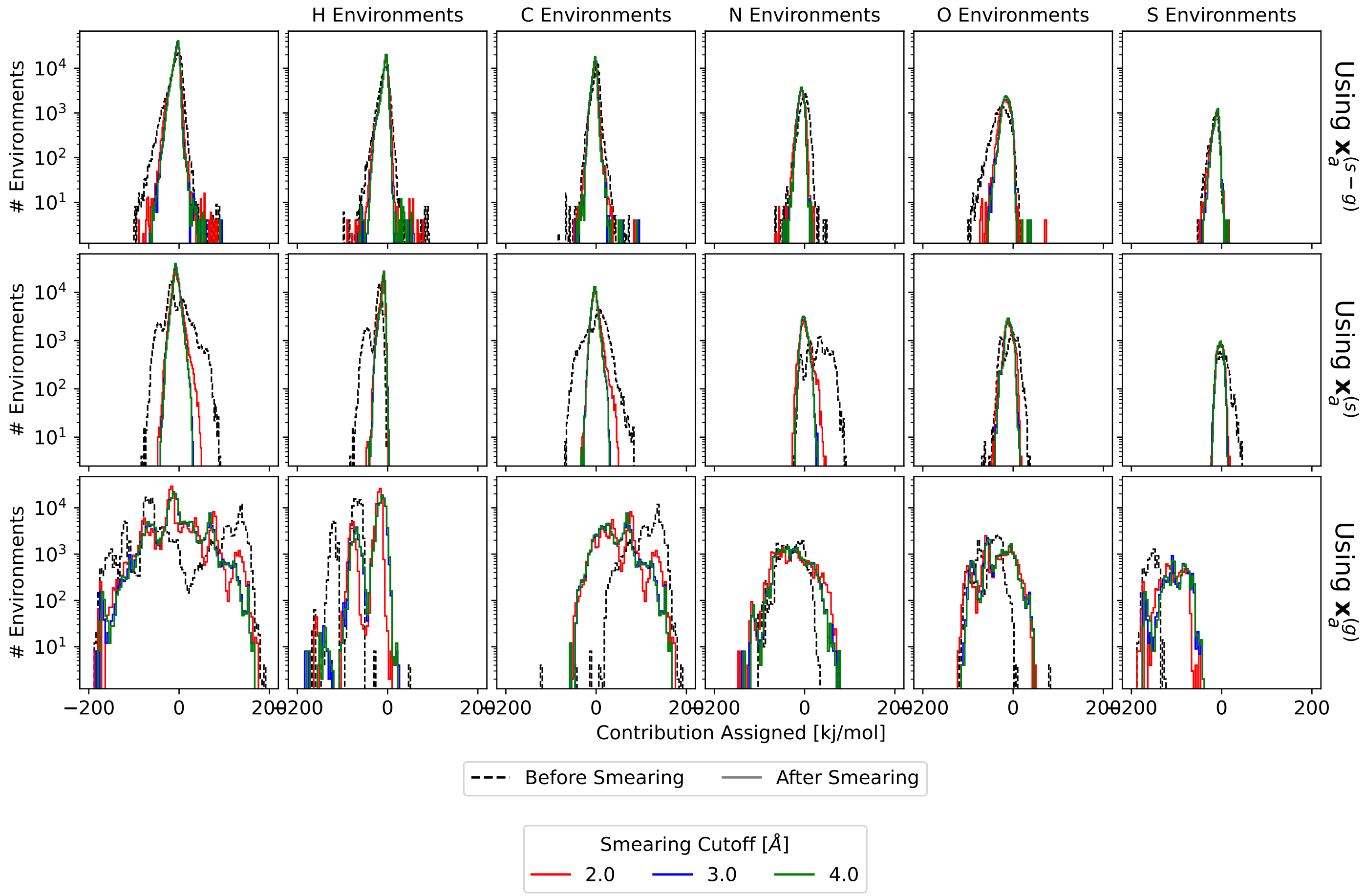

Fig. S2 Effect of "Smearing" the Contributions using Eq. (11). Note that $\mathbf{x}_c^{(s-g)}$ yields the most stable result, compared to $\mathbf{x}_c^{(s)}$ and $\mathbf{x}_c^{(g)}$.

Notice that all of these vary based on representation and environment. In most cases, we see the distribution narrow with the smearing – signifying a regularization to the estimated contributions (*e.g.*, dampening of extreme values). The smearing converges to a constant set of contributions with an increasing smearing cutoff, typically 2.0-3.0 Å (we use 2.0Å for all results in the text).

B.3 Identifying Molecular Motifs

We employed RDKIT Substructure Matching[48] to identify the molecular motifs. For this, we took (our typical) `.xyz` format of the molecular geometries and converted them to `.mol` format using the `openbabel` software[49]. When labeling each collection of atoms, we assign each fragment its most specific designation, *e.g.*, each *nitro* group was not also considered as two *nitroso* groups. We used the SMARTS strings[50] listed in Table S3 to identify motifs in the molecules.

	Name	SMARTS String	# Instances
Sulfur-Based	Disulfide	[#6]~[#16;X2]~[#16;X2]~[#6]	256
	Dithiole	s1~s~c~c~c1, s1~c~s~c~c1	138
	Sulfide Chain	[#16]~[#16;X2]~[#16;X2]~[#16]	200
	Sulfinamide	[SX3](~[OX1])(~[#6])~[#7]	7
	Sulfinyl	[!#8]~[#16;X3](~[!#8])~[OX1]	190
	Sulfonate Esters	[SX4](~[OX1])(~[OX1])([#6])~[O;X2,-]	58
	Sulfonyl	[!#8]~[#16;X4](~[OX1])(~[!#8])~[OX1]	545
	Thiazole	s1~n~c~c~c1, s1~c~n~c~c1	163
	Thiazolidine	S1~N~C~C~C1, S1~C~N~C~C1	138
	Thiocarbonyl	C~[SX1]	359
	Thiocarboxamide	[#1,#6]~C(~[SX1])[#7]	176
	Thiodiazole	s1~n~c~n~c1, s1~c~n~n~c1, s1~n~n~c~c1, s1~n~c~c~n1	289
	Thioether	[#6]~[SX2]~[#6]	1920
	Thioketone	[#6]~[#6](~[S;X1])[#6]	42
	Thiol	[#6;!$(C=,#[!#6])][SX2H]	54
	Thiophene	s1~c~c~c~c1	516
	Thiourea	[#7]~C(~[SX1])[#7]	318
Nitrogen-Based	Aryl Amines	[N$(N-c);H1,H2;r0]	1209
	Carbonyls	[#6,#7,#1]~[C!$([C;X3](~[#8])~[#8])](~[#6,#8,#1])~[O;X1]	1549
	3°Amines	[#7;X3;H0]([#6])([#6])[#6]	1859
	2°Amines	[#7;X3;H1]([#6])[#6]	1504
	1°Amines	[#7;X3;H2][#6]	752
	Acetamide	[#7;X3;r0;H2]~[#6;X3;H0]~[O;X1]	342
	Azide	[#7;X2]~[#7;X2]~[#7;X1]	266
	Azo	[#6]-N=N-[#6]	115
	Carbamide	[#7;X3]~C(~[O;X1])~[#7;X3]	399
	Diazine	n1~n~c~c~c~c1, n1~c~n~c~c~c1, n1~c~c~n~c~c1	758
	Hydrazine	[#7;X3]~[#7;X3;H2]	414
	Hydroxyl- amines	[#6]~[#7;X3](~[#6])~[#8;H1]	92
	Imidazole	n1~c~n~c~c1	444
	Isoxazole	o1~n~c~c~c1, o1~c~n~c~c1	78
	Nitrile	[#6]~[#7;X1]	68
	Nitro	[#8;X1]~[#7]~[#8;X1]	2129
	Nitroso	[#7]~[#8;X1]	498
	Oxidiazole	o1~n~c~n~c1, o1~c~n~n~c1, o1~n~n~c~c1, o1~n~c~c~n1	344
	Oxime	[#6]~[#7;X2]~[#8;H1]	285
	Pentazole	n1~n~n~n~n1	4
	Pyrazole	n1~n~c~c~c1	458
	Pyridine	n1~c~c~c~c~c1	574
	Pyrrole	n1~c~c~c~c1	219
	Tetrazine	n1~n~n~n~c~c1, n1~n~c~n~n~c1	74
	Tetrazole	n1~c~n~n~n1	630
	Triazine	n1~n~n~c~c~c1, n1~n~c~n~c~c1, n1~c~n~c~n~c1	285
	Triazole	n1~n~n~c~c1, n1~c~n~n~c1	657
Oxygen-Based	Carboxyls	[O;X1]~C~[O;H1]	1023
	Alcohols	[#6!$([#6]=O)]~[O;H1]	2603
	Carbonate	[#8;X2]~[C](~[#8;X1])~[#8;X2]	54
	Epoxide	O1CC1	252
	Ester	C(~[O;X1])-[O;H0;X2]-[C]	1218
	Ether	[#8;X2](~[C;r0])~[#6]	736
	Furan	o1~c~c~c~c1	256
	Ketone	[#6][CX3](~[O;X1])[#6]	1041
	Peroxides	[#8;X2]~[O;X2]	77
	Water	[OH2]	868
Carbon-Based	Alkane	[C;H2,H3]-[C;H2,H3]	4784
	Alkene	[C;H1,H0]=[C;H1,H0]	2413
	Alkyne	[C]#[C;H1]	8
	Benzene-like Rings	c1ccccc1	3280
	Ethyl	[C;H2;X4]~[C;H3;X4]	709
	Methyl	[C;H3;X4]	5313

Table S3 Table of SMARTS Strings used to Identify Molecular Motifs. Notice that some SMARTS strings deviate from typical convention in order to accommodate the translation from thermodynamic coordinates (.xyz) to molecular connectivity graph format (.mol), as well as to define certain groups as mutually exclusive.

C Additional Results and Visualizations

C.1 Learning Curves

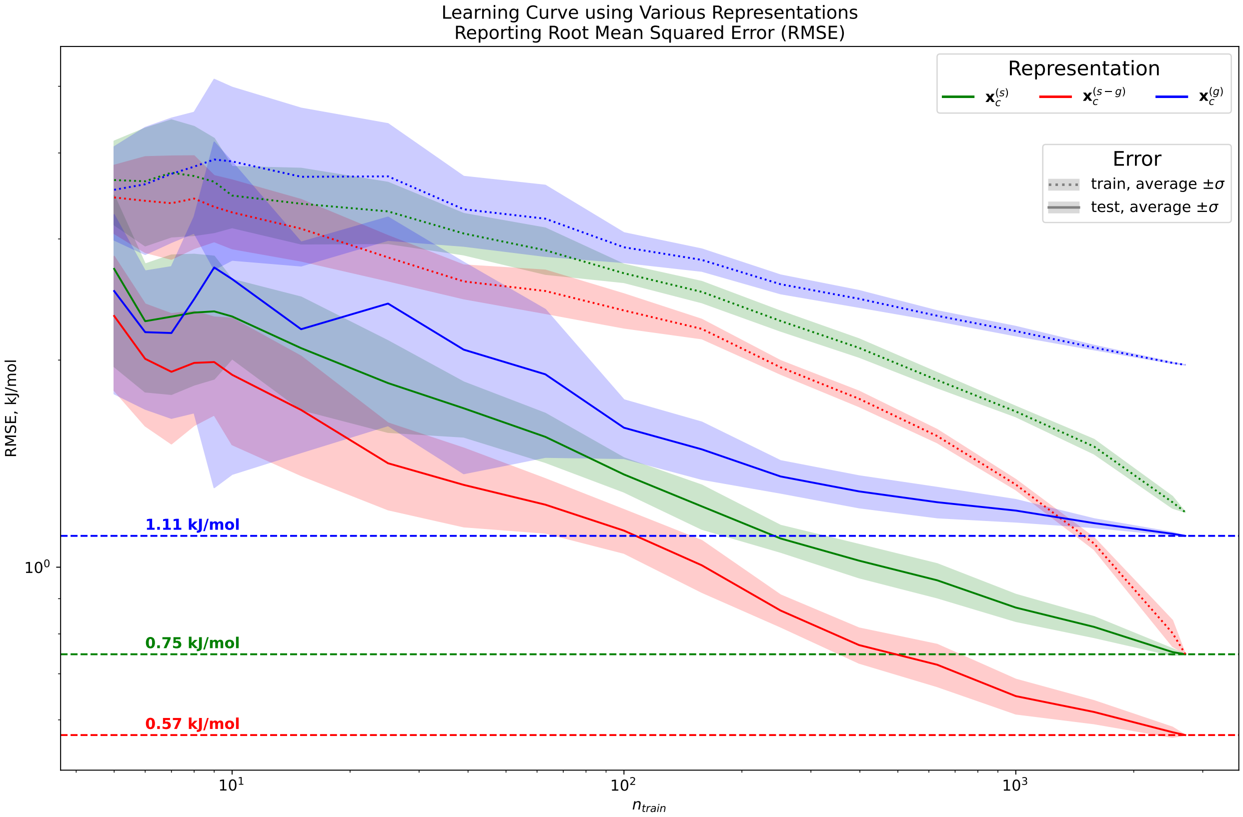

(a) Learning Curve of Various Representations on δ_c.

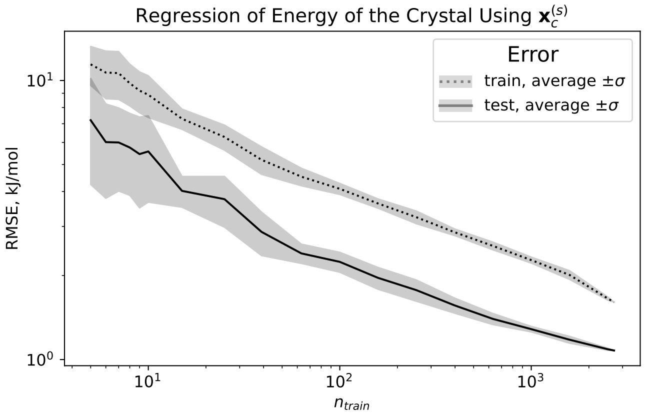

(b) Learning Curve of $\mathbf{x}_c^{(s)}$ on $\mathbf{e}_c$.

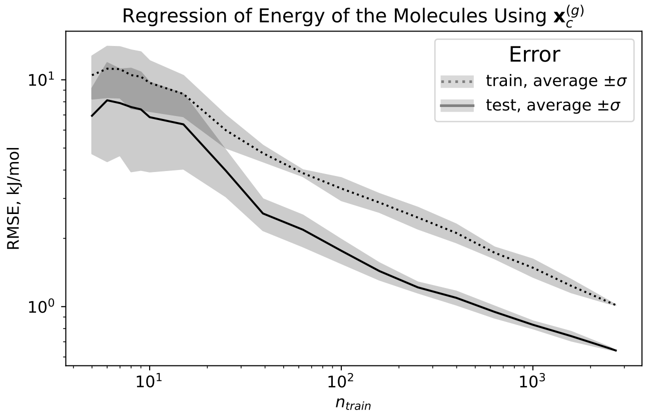

(c) Learning Curve of $\mathbf{x}_m^{(g)}$ on $\mathbf{e}_m$.

Fig. S3 Learning curves for lattice energy for the various representations used in this study. We varied the ratio of mutually-exclusive and randomly-selected training and testing points in each learning curve and conducted a regularized ridge regression. For each ratio, we conducted ten trials with random training sets and report the average (line) and standard deviations (shaded area).

For each descriptor, we constructed learning curves to demonstrate the saturation (or lack thereof) of the regression model on lattice energy. Each learning curve was run using `scikit-learn.model_selection.learning_curve` using a 10-fold cross-validation on a regularized ridge regression pulling randomly from our established training set. All learning curves demonstrate that we are still within a small-data regime (noted by the absence of saturation), justifying our focus on simple and data-efficient linear models.

C.2 Kernel Ridge Regression Models

Kernel Type	Structure-Wise Definition $K_{AB} = \frac{1}{n_A n_B} \sum_{a,b} \cdots$	Equivalent RKHS Definition $K =$
Crystal Environments	$k\left(\mathbf{x}_a^{(s)}, \mathbf{x}_b^{(s)}\right)$	$\left[\phi_c^{(s)} \left(\phi_{c'}^{(s)}\right)^T\right]$
Molecular Environments	$k\left(\mathbf{x}_a^{(g)}, \mathbf{x}_b^{(g)}\right)$	$\left[\phi_c^{(g)} \left(\phi_{c'}^{(g)}\right)^T\right]$
Remnant of the RKHS Features	$k\left(\mathbf{x}_a^{(s)}, \mathbf{x}_b^{(s)}\right) + k\left(\mathbf{x}_a^{(g)}, \mathbf{x}_b^{(g)}\right)$ $-k\left(\mathbf{x}_a^{(s)}, \mathbf{x}_b^{(g)}\right) - k\left(\mathbf{x}_a^{(g)}, \mathbf{x}_b^{(s)}\right)$	$\left[\left(\phi_c^{(s)} - \phi_c^{(g)}\right)\left(\phi_{c'}^{(s)} - \phi_{c'}^{(g)}\right)^T\right]$
Remnant Environments	$k\left(\mathbf{x}_a^{(s-g)}, \mathbf{x}_b^{(s-g)}\right)$	$\left[\phi_c^{(s-g)} \left(\phi_{c'}^{(s-g)}\right)^T\right]$

Table S4 Equations for Non-linear Kernels We computed regressions using the given kernel equations for crystals A and B and their corresponding atoms a and b. Mercer's theorem ensures that for a positive-definite kernel on x, there exists a non-linear mapping $x \mapsto \phi$ such that $K = \phi\phi^T$, for which we have included our notation.

We computed regressions with both (i) an optimized RBF kernel (with optimal γ found by employing a subset of 1,000 crystals taken from the training set and (ii) a parameter-free cosine kernel. We ran kernel ridge regression models using `scikit-learn.linear_model.KernelRidge`[51] employing a 90/10 training / validation split. The results on the offset test set are given in Table S5.

Regression Equation	RBF Kernel RMSE	RBF Kernel MAE	Cosine Kernel RMSE	Cosine Kernel MAE
$\mathbf{e}_c = \left[\phi_c^{(s)} \left(\phi_{c'}^{(s)}\right)^T\right] \mathbf{w}_c$	0.904	0.665	1.039	0.772
$\mathbf{e}_m = \left[\phi_m^{(g)} \left(\phi_{m'}^{(g)}\right)^T\right] \mathbf{w}_m$	0.392	0.294	0.496	0.373
$\delta_c = \left[\phi_c^{(s)} \left(\phi_{c'}^{(s)}\right)^T\right] \mathbf{w}_c - \sum_{m \in c} \frac{n_m}{n_c} \left(\left[\phi_m^{(g)} \left(\phi_{m'}^{(g)}\right)^T\right] \mathbf{w}_m\right)$	0.913	0.681	1.061	0.805
$\delta_c = \left[\phi_c^{(s)} \left(\phi_{c'}^{(s)}\right)^T\right] \mathbf{w}$	0.694	0.508	0.742	0.526
$\delta_c = \left[\phi_c^{(g)} \left(\phi_{c'}^{(g)}\right)^T\right] \mathbf{w}$	1.097	0.714	1.097	0.71
$\delta_c = \left[\left(\phi_c^{(s)} - \phi_c^{(g)}\right)\left(\phi_{c'}^{(s)} - \phi_{c'}^{(g)}\right)^T\right] \mathbf{w}$	0.541	0.403	0.589	0.425
$\delta_c = \left[\phi_c^{(s-g)} \left(\phi_{c'}^{(s-g)}\right)^T\right] \mathbf{w}$	0.515	0.382	0.66	0.489

Table S5 Results of Kernel Ridge Regression Exercises. We have written each equation using reproducing kernel hilbert space (RKHS) notation (see Table S4), denoting the training set with $'$. Note the difference between the bottom entries, which represent (1) the "remnant version of non-linear feature vectors ϕ and (2) the non-linear mapping of the remnant vector employed in the main text. In each kernel regression, an independent, 2-fold cross-validated model was built on our 2'707 crystal training set in an 80/20 train/validation split. Here we report the RMSE and MAE (in kJ/mol) on a separate testing set of 551 crystals (or the coinciding 628 molecules). Each regression equation $\mathbf{w}$ is unique to that regression.

C.3 Expanded Violin Plot

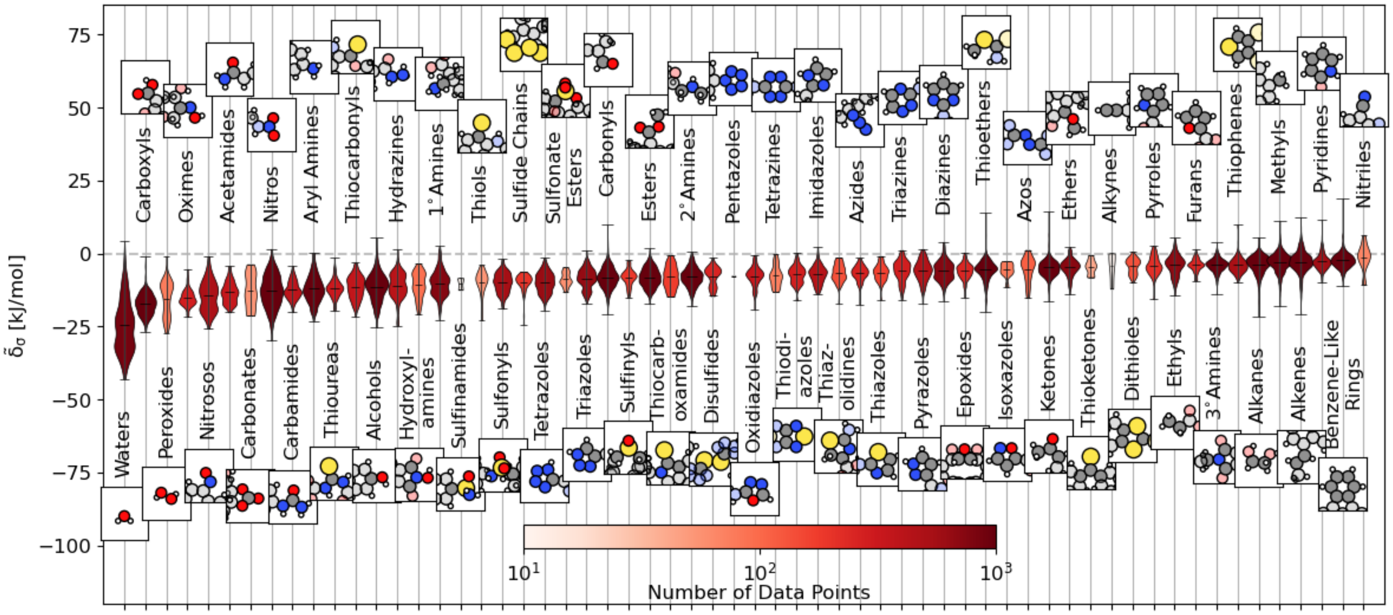

Fig. S4 Violin Plot from Fig. 2, expanded to include all functional groups. A representative example is shown above or below the violin plot with the functional group highlighted for each functional group. The lines on each plot denote each group's extrema and mean contributions. The plots colors reflect the number of examples within the dataset, ranging from 4 (pentazole) to 5313 (methyl groups)

.

C.4 Images of Phenanthrene Polymorphs

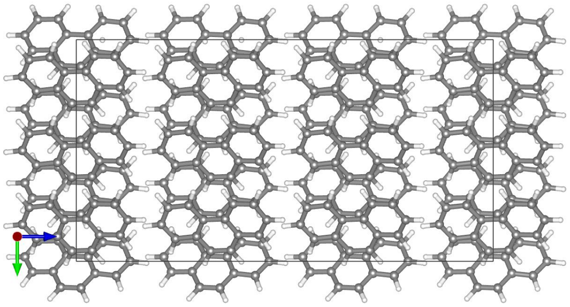

(a) View of PHENAN08[52] along a axis.

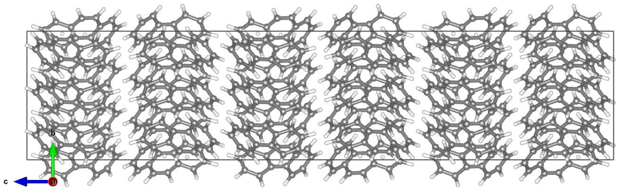

(b) View of PHENAN14[15] along a axis.

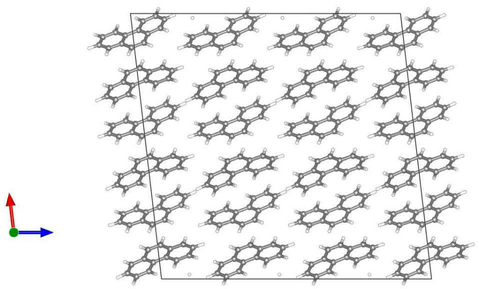

(c) View of PHENAN08[52] along b axis.

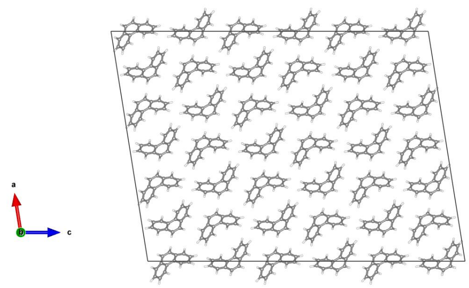

(d) View of PHENAN14[15] along b axis.

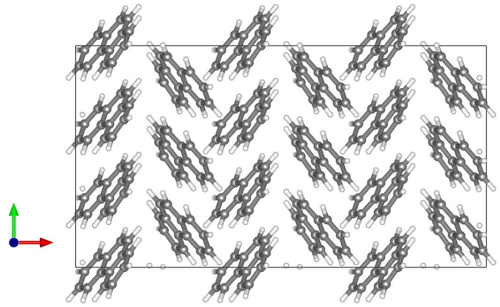

(e) View of PHENAN08[52] along c axis.

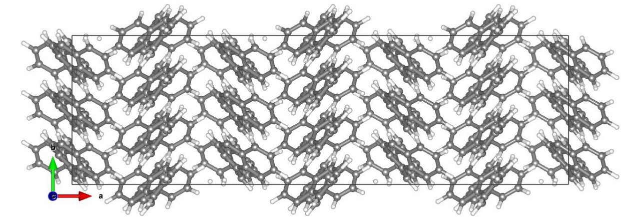

(f) View of PHENAN14[15] along c axis.

Fig. S5 Axial views of the stable (a, c, e) and unstable (b, d, f) phenanthrene polymorphs. 3x3x3 supercells are shown to include all intermolecular interactions.

C.5 Additional PCovR Maps

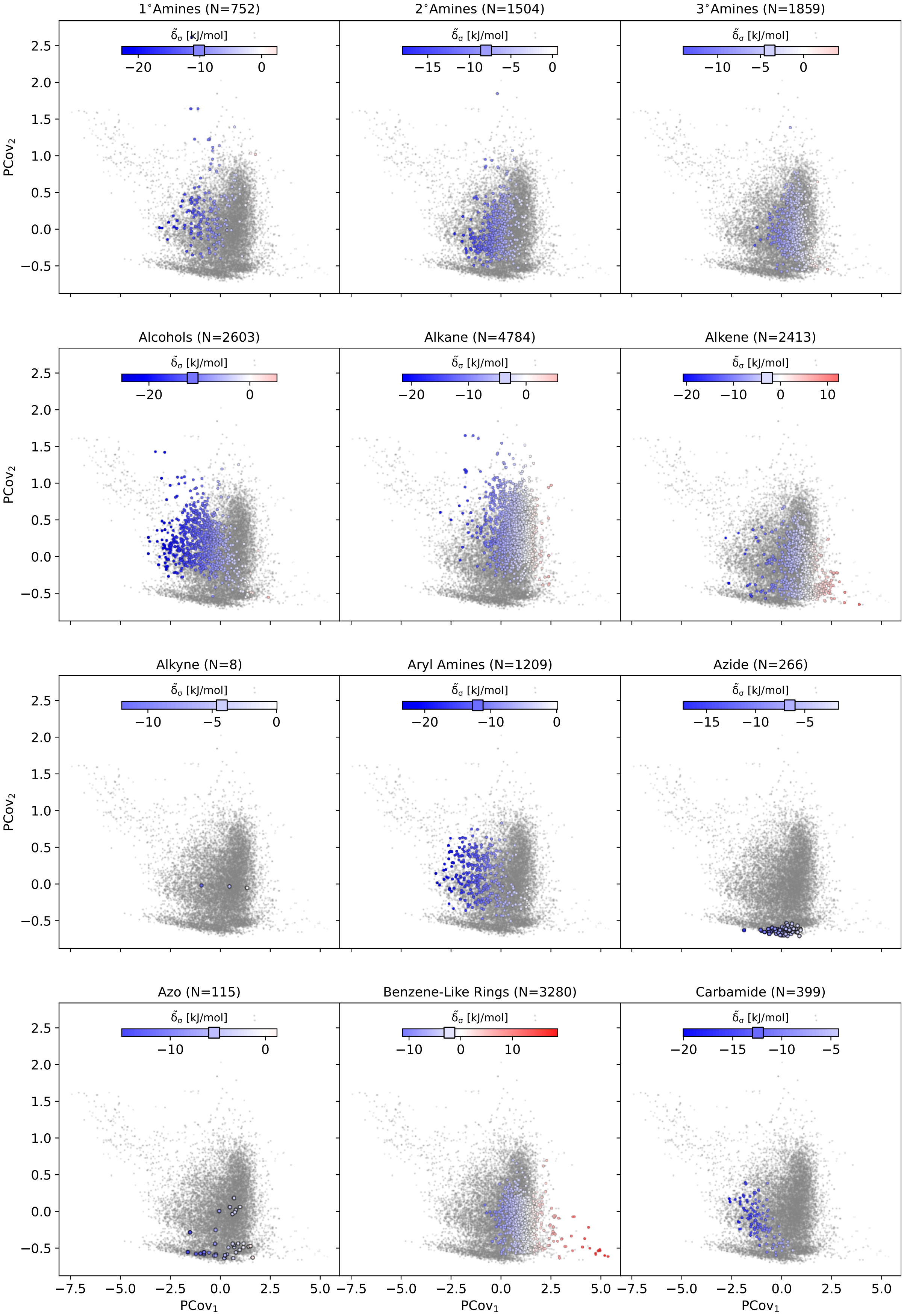

Fig. S6 PCovR map from Fig. 3, highlighting each type of functional group. Each map is on the same color scale; however, we have truncated the color bar to demonstrate the range of cohesive interactions. We have denoted the average of all group members by a square marker on the color bar.

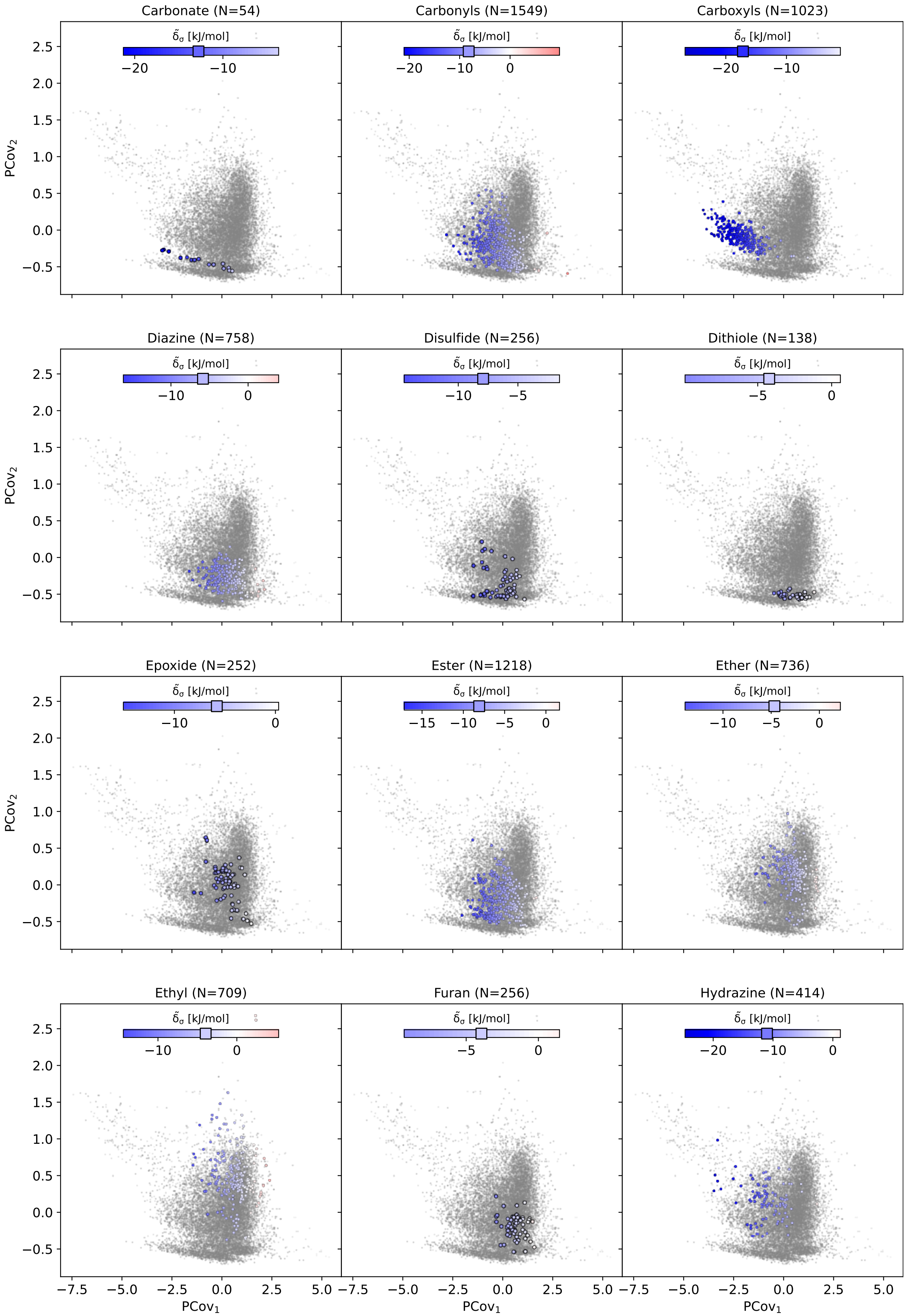

Fig. S7 PCovR map from Fig. 3, highlighting each type of functional group. Each map is on the same color scale; however, we have truncated the color bar to demonstrate the range of cohesive interactions. We have denoted the average of all group members by a square marker on the color bar.

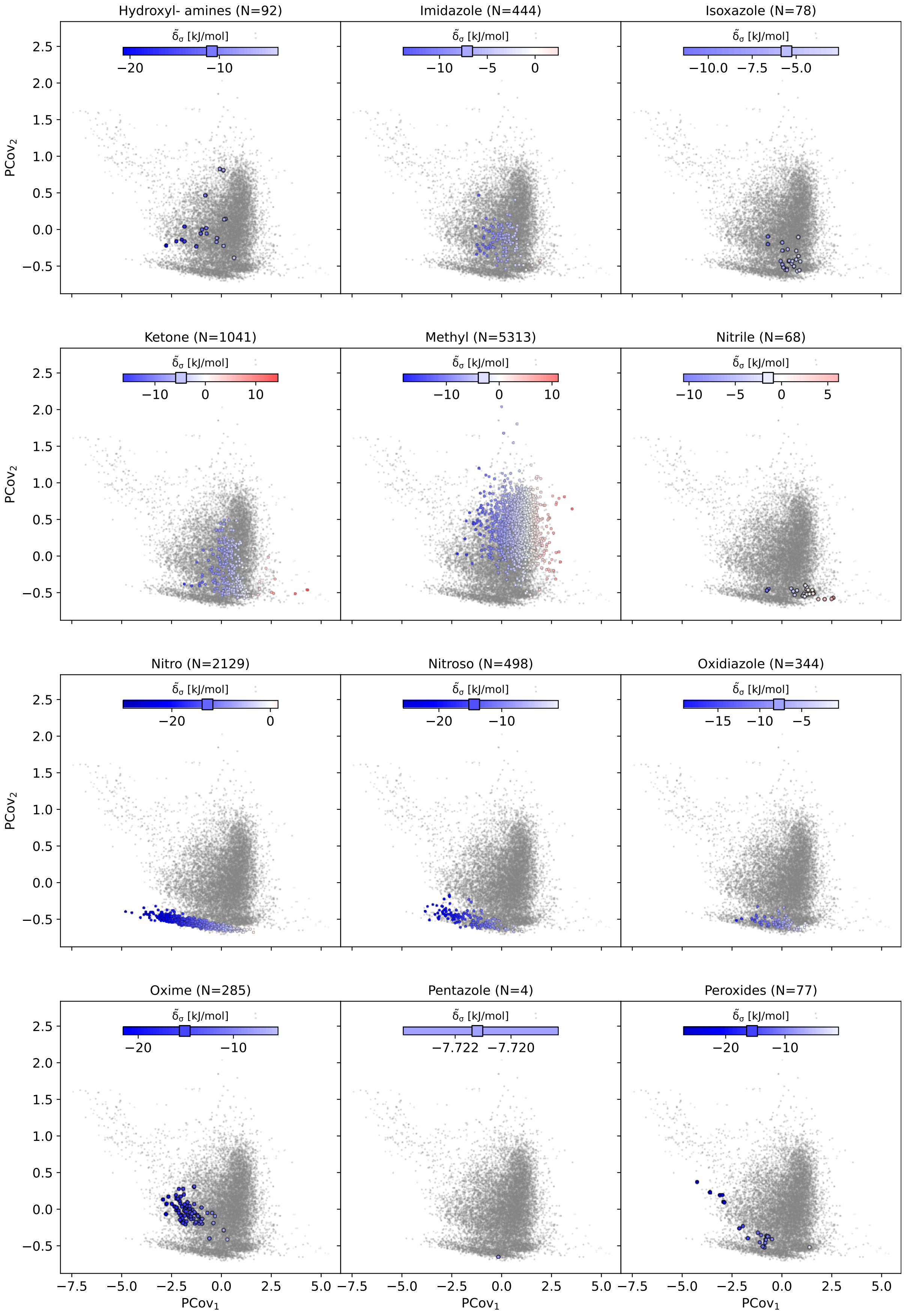

Fig. S8 PCovR map from Fig. 3, highlighting each type of functional group. Each map is on the same color scale; however, we have truncated the color bar to demonstrate the range of cohesive interactions. We have denoted the average of all group members by a square marker on the color bar.

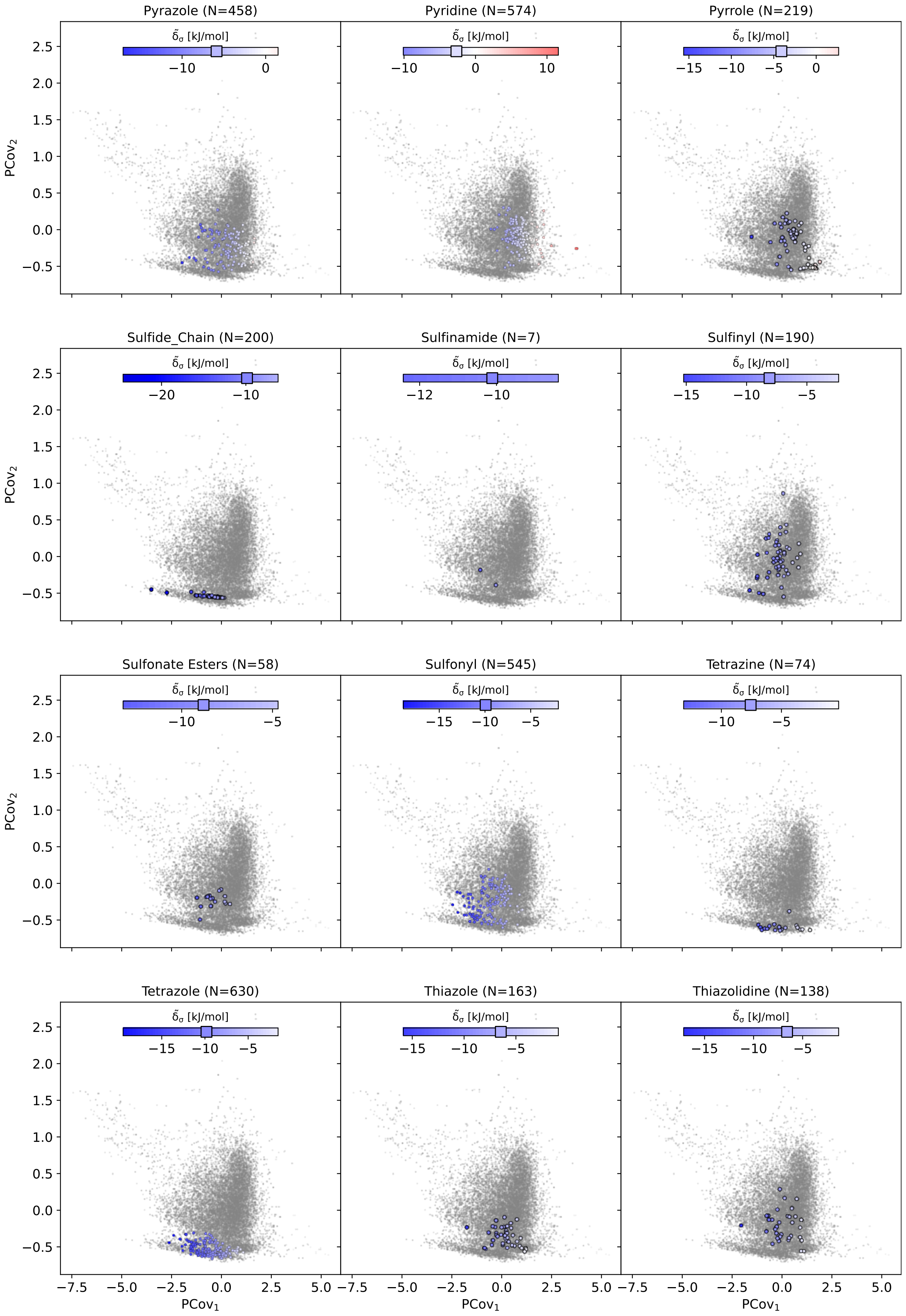

Fig. S9 PCovR map from Fig. 3, highlighting each type of functional group. Each map is on the same color scale; however, we have truncated the color bar to demonstrate the range of cohesive interactions. We have denoted the average of all group members by a square marker on the color bar.

C.6 Parity Plot for Ethenzamide Dataset

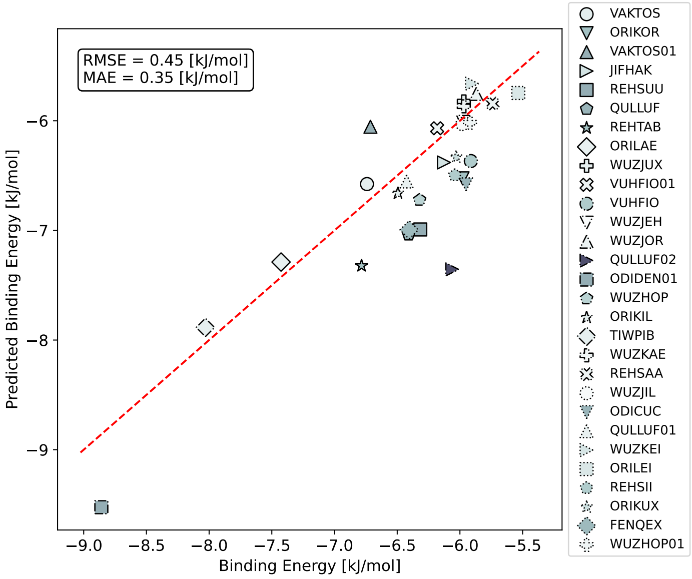

Fig. S10 Parity plot showing regression errors for the 29 ethenzamide co-crystals. Regressions were computed using the regularized ridge regression trained the remnant descriptor $\mathbf{x}_c^{(s-g)}$ of the 2'707 training set crystals. Here we have labeled each point by its corresponding CSD refcode.

Notes and references

1 M. Cordova, E. A. Engel, A. Stefaniuk, F. Paruzzo, A. Hofstetter, M. Ceriotti and L. Emsley, *The Journal of Physical Chemistry C*, 2022, **126**, 16710–16720.

2 C. S. Adorf, V. Ramasubramani, B. D. Dice, M. M. Henry, P. M. Dodd and S. C. Glotzer, *glotzerlab/signac*, 2019, https://doi.org/10.5281/zenodo.2581326.

3 C. S. Adorf, P. M. Dodd, V. Ramasubramani and S. C. Glotzer, *Computational Materials Science*, 2018, **146**, 220–229.

4 B. Hourahine, B. Aradi, V. Blum, F. Bonafé, A. Buccheri, C. Camacho, C. Cevallos, M. Y. Deshaye, T. Dumitric, A. Dominguez, S. Ehlert, M. Elstner, T. van der Heide, J. Hermann, S. Irle, J. J. Kranz, C. Köhler, T. Kowalczyk, T. Kuba, I. S. Lee, V. Lutsker, R. J. Maurer, S. K. Min, I. Mitchell, C. Negre, T. A. Niehaus, A. M. N. Niklasson, A. J. Page, A. Pecchia, G. Penazzi, M. P. Persson, J. ezá, C. G. Sánchez, M. Sternberg, M. Stöhr, F. Stuckenberg, A. Tkatchenko, V. W.-z. Yu and T. Frauenheim, *The Journal of Chemical Physics*, 2020, **152**, 124101.

5 M. Gaus, A. Goez and M. Elstner, *Journal of Chemical Theory and Computation*, 2013, **9**, 338–354.

6 M. Gaus, X. Lu, M. Elstner and Q. Cui, *Journal of Chemical Theory and Computation*, 2014, **10**, 1518–1537.

7 P. Giannozzi, S. Baroni, N. Bonini, M. Calandra, R. Car, C. Cavazzoni, D. Ceresoli, G. L. Chiarotti, M. Cococcioni, I. Dabo, A. Dal Corso, S. de Gironcoli, S. Fabris, G. Fratesi, R. Gebauer, U. Gerstmann, C. Gougoussis, A. Kokalj, M. Lazzeri, L. Martin-Samos, N. Marzari, F. Mauri, R. Mazzarello, S. Paolini, A. Pasquarello, L. Paulatto, C. Sbraccia, S. Scandolo, G. Sclauzero, A. P. Seitsonen, A. Smogunov, P. Umari and R. M. Wentzcovitch, *Journal of Physics: Condensed Matter*, 2009, **21**, 395502.

8 J. Perdew, K. Burke and M. Ernzerhof, *Physical Review Letters*, 1996, **77**, 3865–3868.

9 S. Grimme, *Journal of Computational Chemistry*, 2006, **27**, 1787–1799.

10 A. Dal Corso, *Computational Materials Science*, 2014, **95**, 337–350.

11 G. Kresse and D. Joubert, *Phys. Rev. B*, 1999, **59**, 1758–1775.

12 G. J. Martyna and M. E. Tuckerman, *The Journal of Chemical Physics*, 1999, **110**, 2810–2821.

13 Marelli, E. and Casati, N. and Gozzo, F. and Macchi, P. and Simoncic, P. and Sironi, A., *CrystEngComm*, 2011, **13**, 6845.

14 Podsiadlo, M. and Olejniczak, A. and Katrusiak, A., *The Journal of Physical Chemistry C*, 2013, **117**, 4759.

15 Fabbiani, F.P.A. and Allan, D.R. and David, W.I.F. and Moggach, S.A. and Parsons, S. and Pulham, C.R., *CrystEngComm*, 2004, **6**, 504.

16 Rheingold, A.L., *CSD Communication*, 2016.

17 Adam, M.S. and Gutmann, M.J. and Leech, C.K. and Middlemiss, D.S. and Parkin, A. and Thomas, L.H. and Wilson, C.C., *New Journal of Chemistry*, 2010, **34**, 85.

18 Breton, G.W. and Martin, K.L., *Journal of Organic Chemistry*, 2002, **67**, 6699.

19 Talberg, H.J., *Acta Chemica Scandinavica [1989-1999]*, 1977, **31**, 485.

20 Castello-Mico, A. and Julia Nafe, and Higashida, K. and Karaghiosoff, K. and Gingras, M. and Knochel, P., *Organic Letters*, 2017, **19**, 360.

21 Ohkita, M. and Suzuki, T. and Nakatani, K. and Tsuji, T., *Chemistry Letters*, 2001, 988.

22 K.Mitsudo, T.Murakami, T.Shibasaki, T.Inada, H.Mandai, H.Ota, Seiji Suga, *Synlett*, 2016, **27**, 2327.

23 Chien-Yang Chiu, and Bumjung Kim, and Gorodetsky, A.A. and Sattler, W. and Sujun Wei, and Sattler, A. and Steigerwald, M. and Nuckolls, C., *Chemical Science*, 2011, **2**, 1480.

24 Mereiter, K. and Rosenau, T., *CSD Communication*, 2004.

25 Berridge, R. and Serebryakov, I.M. and Skabara, P.J. and Orti, E. and Viruela, R. and Pou-Amerigo, R. and Coles, S.J. and Hursthouse, M.B., *Journal of Materials Chemistry*, 2004, **14**, 2822.

26 Britton, D., *Acta Crystallographica Section E: Structure Reports Online [2001-2014]*, 2002, **58**, o637.

27 Maris, T., *CSD Communication*, 2016.

28 Panfilova, L.V. and Antipin, M.Yu. and Churkin, Yu.D. and Struchkov, Yu.T., *Khimiya Geterotsiklicheskikh Soedinenii*, 1979, 1201.

29 Lincke, G. and Finzel, H.-U., *Crystal Research and Technology*, 1996, **31**, 441.

30 Fang-Ming Miao, and Jin-Ling Wang, and Xiu-Shen Miao, ., *Acta Crystallographica,Section C: Crystal Structure Communications [1983-2014]*, 1995, **51**, 712.

31 Rabaca, S. and Oliveira, S. and Cerdeira, A.C. and Simao, D. and Santos, I.C. and Almeida, M., *Tetrahedron Letters*, 2014, **55**, 6992.

32 Morak, B. and Pluta, K. and Suwinska, K. and Grymel, M. and Besnard, C. and Schiltz, M. and Kloc, C. and Siegrist, T., *Heterocycles*, 2005, **65**, 2619.

33 Entwistle, R.F. and Iball, J. and Motherwell, W.D.S. and Thompson, B.P., *Acta Crystallographica,Section B:Struct.Crystallogr.Cryst.Chem. [1968-1982]*, 1969, **25**, 770.

34 Derissen, J.L. and Timmermans, C. and Schoone, J.C., *Crystal Structure Communications*, 1979, **8**, 533.

35 Newkome, G.R. and Joo, Y.J. and Theriot, K.J. and Fronczek, F.R., *Journal of the American Chemical Society*, 1986, **108**, 6074.

36 Aitipamula, S. and Pui Shan Chow, and Tan, R.B.H., *CrystEngComm*, 2010, **12**, 3691.

37 Hariprasad, V.M. and Nechipadappu, S.K. and Trivedi, D.R., *Crystal Growth and Design*, 2016, **16**, 4473.

38 Khatioda, R. and Bora, P. and Sarma, B., *Crystal Growth and Design*, 2018, **18**, 4637.

39 Aitipamula, S. and Wong, A.B.H. and Pui Shan Chow ,and Tan, R.B.H., *CrystEngComm*, 2012, **14**, 8515.

40 Aitipamula, S. and Pui Shan Chow, and Tan, R.B.H., *CrystEngComm*, 2009, **11**, 1823.

41 Aitipamula, S. and Pui Shan Chow,and Tan, R.B.H., *Crystal Growth and Design*, 2010, **10**, 2229.

42 Aitipamula, S. and Pui Shan Chow,and Tan, R.B.H., *CrystEngComm*, 2009, **11**, 889.

43 Khatioda, R. and Saikia, B. and Das, P.J. and Sarma, B., *CrystEngComm*, 2017, **19**, 6992.

44 Kozak, A. and Marek, P.H. and Pindelska, E., *Journal of Pharmaceutical Sciences*, 2018, **108**, 1476.

45 Sarmah, K.K. and Boro, K. and Arhangelskis, M. and Thakuria, R., *CrystEngComm*, 2017, **19**, 826.

46 F. Musil, M. Veit, A. Goscinski, G. Fraux, M. J. Willatt, M. Stricker, T. Junge and M. Ceriotti, *The Journal of Chemical Physics*, 2021, **154**, 114109.

47 M. J. Willatt, F. Musil and M. Ceriotti, *Phys. Chem. Chem. Phys.*, 2018, **20**, 29661–29668.

48 *RDKit: Open-source cheminformatics.*, `https://www.rdkit.org`.

49 N. M. O'Boyle, M. Banck, C. A. James, C. Morley, T. Vandermeersch and G. R. Hutchison, *Journal of Cheminformatics*, 2011, **3**, 33.

50 *SMARTS: A Language for Describing Molecular Patterns*, 1997, `daylight.com/dayhtml/doc/theory/theory.smarts.html`.

51 F. Pedregosa, G. Varoquaux, A. Gramfort, V. Michel, B. Thirion, O. Grisel, M. Blondel, P. Prettenhofer, R. Weiss, V. Dubourg, J. Vanderplas, A. Passos, D. Cournapeau, M. Brucher, M. Perrot and E. Duchesnay, *Journal of Machine Learning Research*, 2011, **12**, 2825–2830.
52 Petricek, V. and Cisarova, I. and Hummel, L. and Kroupa, J. and Brezina, B., *Acta Crystallographica,Section B: Structural Science [1983-2012]*, 1990, **46**, 830.
